# Pathogen-Mediated Assembly of Plant-Beneficial Bacteria to Alleviate *Fusarium* Wilt in *Pseudostellaria heterophylla*

**DOI:** 10.3389/fmicb.2022.842372

**Published:** 2022-03-30

**Authors:** Qing-Song Yuan, Lu Wang, Hui Wang, Xiaoai Wang, Weike Jiang, Xiaohong Ou, Chenghong Xiao, Yanping Gao, Jiao Xu, Ye Yang, Xiuming Cui, Lanping Guo, Luqi Huang, Tao Zhou

**Affiliations:** ^1^Resource Institute for Chinese and Ethnic Materia Medica, Guizhou University of Traditional Chinese Medicine, Guiyang, China; ^2^Faculty of Life Science and Technology, Kunming University of Science and Technology, Kunming, China; ^3^National Resource Center for Chinese Materia Medica, China Academy of Chinese Medical Sciences, Beijing, China

**Keywords:** microbial community, *Fusarium* wilt, *F. oxysporum*, *P. poae*, *P. heterophylla*

## Abstract

*Fusarium* wilt (FW) is a primary replant disease that affects *Pseudostellaria heterophylla* (Taizishen) and is caused by *Fusarium oxysporum*, which occurs widely in China under the continuous monocropping regime. However, the ternary interactions among the soil microbiota, *P. heterophylla*, and *F. oxysporum* remain unknown. We investigated the potential interaction relationship by which the pathogen-mediated *P. heterophylla* regulates the soil and the tuberous root microbiota *via* high-throughput sequencing. Plant–pathogen interaction assays were conducted to measure the arrival of *F. oxysporum* and *Pseudomonas poae* at the tuberous root *via* qPCR and subsequent seedling disease incidence. A growth assay was used to determine the effect of the tuberous root crude exudate inoculated with the pathogen on *P. poae*. We observed that pathogen-mediated *P. heterophylla* altered the diversity and the composition of the microbial communities in its rhizosphere soil and tuberous root. Beneficial microbe *P. poae* and pathogen *F. oxysporum* were significantly enriched in rhizosphere soil and within the tuberous root in the FW group with high severity. Correlation analysis showed that, accompanied with FW incidence, *P. poae* co-occurred with *F. oxysporum*. The aqueous extract of *P. heterophylla* tuberous root infected by *F. oxysporum* substantially promoted the growth of *P. poae* isolates (H1-3-A7, H2-3-B7, H4-3-C1, and N3-3-C4). These results indicated that the extracts from the tuberous root of *P. heterophylla* inoculated with *F. oxysporum* might attract *P. poae* and promote its growth. Furthermore, the colonization assay found that the gene copies of *sucD* in the *P. poae* and *F. oxysporum* treatment (up to 6.57 × 10^10^) group was significantly higher than those in the *P. poae* treatment group (3.29 × 10^10^), and a pathogen-induced attraction assay found that the relative copies of *sucD* of *P. poae* in the *F. oxysporum* treatment were significantly higher than in the H_2_O treatment. These results showed that *F. oxysporum* promoted the colonization of *P. poae* on the tuberous root *via F. oxysporum* mediation. In addition, the colonization assay found that the disease severity index in the *P. poae* and *F. oxysporum* treatment group was significantly lower than that in the *F. oxysporum* treatment group, and a pathogen-induced attraction assay found that the disease severity index in the *F. oxysporum* treatment group was significantly higher than that in the H_2_O treatment group. Together, these results suggest that pathogen-mediated *P. heterophylla* promoted and assembled plant-beneficial microbes against plant disease. Therefore, deciphering the beneficial associations between pathogen-mediated *P. heterophylla* and microbes can provide novel insights into the implementation and design of disease management strategies.

## Introduction

*Pseudostellaria heterophylla* (Crown Prince Ginseng/Taizishen) belongs to the Caryophyllaceae family and has been one of China’s most widely used traditional herbal medicines for over 3,000 years. It is mainly produced and cultivated in a geo-authentic production zone in China’s Guizhou, Anhui, Shandong, and Fujian provinces (Zhao et al., 2016). The herb is widely used for treating diseases that occur in the lung and spleen (Pang et al., 2011). Continuous monoculture of traditional herbal medicines often leads to soil-borne disease. Like other traditional herbal medicines and crops, *P. heterophylla* is replanted on the same field with asexual propagation for many years, resulting in the high incidence of *Fusarium* wilt (FW), a replant disease caused by infection with *Fusarium oxysporum* (Wu et al., 2016b,2020a,^2021^; Chen et al., 2021). FW is characterized by damping-off, wilting, and dry brown rot and is a disastrous disease in many crops (i.e., wheat, cotton, and chickpea) and fruits (i.e., banana, melon, and watermelon). The annual incidence of FW is approximately 20%, leading to a severe reduction in yield and no harvest in extreme cases. In addition, the fumonisin and zearalenone mycotoxins produced by *F. oxysporum* contaminated *P. heterophylla* tuberous root (used for herbal medicine decoction pieces), severely affecting the quality of *P. heterophylla*.

Many previous studies have shown that continuous monocropping of *P. heterophylla* decreases the diversity and alters the composition of the soil microbial community. For example, consecutive cropping of *P. heterophylla* leads to a high incidence of replant disease *via* an imbalanced microbial structure with a higher ratio of pathogens/beneficial bacteria in the rhizosphere soil (Wu et al., 2016a; Zhao et al., 2016). Some studies have also reported that agricultural practices and soil remediation are used to alleviate continuous cropping obstacles. An analysis by Wu et al. (2016b) showed that the application of novel bio-organic fertilizer effectively suppressed FW by enriching the antagonistic bacteria and enhancing the bacterial diversity. Wu et al. (2020a,b) found that *P. heterophylla* resisted replanting disease, or the disease was alleviated by enriching plant-beneficial microbes in the soil and reducing the accumulation of soil-borne pathogens *via* agricultural practices such as growing a natural forest cover or performing intraspecific intercropping. All these studies only found that continuous cropping increased soil-borne diseases by enriching pathogens and reducing beneficial bacteria in the rhizosphere soil of *P. heterophylla*. However, the regulation mechanism of *P. heterophylla* on rhizosphere microorganisms when it suffered from replant disease has not been reported yet.

Soil microorganisms are essential regulators of plant disease resistance, growth, development, fitness, and immunity. In turn, plants regulate their growth and motility under biotic stress or abiotic stress (Chakraborty and Newton, 2011; Liu et al., 2019b,^2020^). Continuous monoculture leads to many soil-borne pathogens in the soil and a high biotic stress environment for plants, which leads to an increased incidence of disease (Lapsansky et al., 2016; Zhang et al., 2016; Kong et al., 2019; Liu et al., 2019a). In recent years, many researchers have found that the enrichment of beneficial microbes in the soil mediated by pathogen infection can alleviate soil-borne disease and expand plant defensive capabilities (Elad et al., 2010). It is unknown whether pathogens can intervene in *P. heterophylla* to enrich beneficial microbes under the continuous monocropping regime.

Many studies have described the disease-induced plant protection mechanism known as the “cry for help” that indirectly contributes to the enrichment of beneficial microbes. When a plant is subjected to nutritional deficiency, its roots secrete amino acid (i.e., asparagine, ornithine, and tryptophan) (Carvalhais et al., 2015; Lebeis et al., 2015) and aromatic organic acids (i.e., nicotine and cinnamic acid) (Zhalnina et al., 2018) to attract specific taxa that can alleviate soil-borne disease. Recent studies also found that the beneficial microbes dynamically respond to the presence of a pathogen, which supports the hypothesis that plants actively assemble specific microbial species or microbial communities to fight soil-borne diseases upon pathogen infection (Berendsen et al., 2018; Lee et al., 2020; Liu et al., 2020).

For example, Arabidopsis promoted the proliferation of three specific bacteria (e.g., *Xanthomonas* sp., *Stenotrophomonas* sp., and *Microbacterium* sp.) in the rhizosphere upon leaf infection with the downy mildew pathogen *Hyaloperonospora arabidopsidis* (Berendsen et al., 2018). Changes in the composition and enrichment of potentially beneficial microorganisms have also been observed in barley roots infected with *F. graminearum* (Deshmukh and Kogel, 2007). The phenomenon also exists in many traditional herbal medicines such as *P. heterophylla* infected with *Serratia marcescens* (Zhang et al., 2016) and *Panax ginseng* (Liu et al., 2019a) infected with *F. oxysporum*. The capacity of plants to exploit protective benefits from their root microbiome is plant species- and genotype-dependent (Elad et al., 2010; Berendsen et al., 2018). Therefore, it is unknown whether *P. heterophylla* as a medicinal plant can recruit specific microbiota to resist diseases. Hence, we think that *P. heterophylla* may recruit specific beneficial microbiota to alleviate diseases *via* a “cry for help” hypothesis. To achieve our aims, (1) we conducted a field experiment whereby a continuous monoculture of *P. heterophylla* was planted in a field for 3 years with high *F. oxysporum* stress. After 4 months, we collected rhizosphere soil (RS), root zone soil (RZ), bulk soil (BK), and tuberous roots of *P. heterophylla* with naturally high (H), low (L), and no (N) symptom of FW. (2) We aimed to investigate how *P. heterophylla* regulates the microbiota in RS, RZ, and tuberous roots infected with FW *via* high-throughput sequencing. (3) Growth assays and plant-pathogen interaction assays further indicated that *P. heterophylla* can attract and assemble specific microorganisms under biotic stress.

## Materials and Methods

### Field Experiments

Field experiments were conducted in 2018 on *P. heterophylla* that was planted on December 13 in major cultivation areas in Shibing County (27°4’21”N, 108°8’0”E, and 759 m a.s.l.) in Guizhou Province, China. The monthly average precipitation is 14–278 mm, and the monthly means temperature is 4.7–27.3°C in Shibing County. For 3 years, monocultured *P. heterophylla* was planted in the experimental field, and there was a widespread occurrence of FW during the last planting. The field was approximately 30 m long and 20 m wide and had 30 ridges 30–40 cm high parallel to the wide side (Supplementary Figure 1A) and two adjacent ridges spaced 20 cm apart. The *P. heterophylla* was planted with a 5- to 10-cm spacing between plants. Shitai 1#, the cultivar studied here, is susceptible to FW.

### Sampling

The two soil-root system compartments of each plant, including RS and RZ, were selected to fully explore the relationship between the soil microbiome and FW incidence in the soil-root system and bulk soil (as a control) (Supplementary Figure 1). Samples were collected on April 13, 2019 (4 months after planting). All plants were randomly chosen with the same disease severity, and the population density, appearance, plant size, growth rate, and growth period were consistent; plants with pest infestations or mechanical damage and those impacted by marginal effects were excluded.

*Fusarium* wilt severity was evaluated as described by Naruto et al. (1991) using a rating scale ranging from 0 to 5: 0, no symptom; 1, 0.1–20.0% withering; 2, 20.1–40.0% withering; 3, 40.1–60.0% withering; 4, 60.1–80.0% withering; and 5, 80.1–100% withering; the coverage range was evaluated based on simple measurements with a grid ruler. Disease severity index (DSI) was calculated by the following formula: Σ (number of samples per rating × rating value)/the sum of samples. Eighteen plants were selected according to FW severity, six plants were grouped and identified as H (FW severity rank ≥ 3), and six plants were grouped and identified as L (FW severity rank = 1–2). Six plants were grouped and identified as N (FW severity rank = 0) (Supplementary Figure 1B).

To accurately evaluate the relationship between the soil microbiome and FW severity, the RS was considered to be the soil tightly attached to tuberous roots, which was collected using a modification of the method previously described by Shi et al. (2019) and Yuan et al. (2020b). In brief, the plant was carefully harvested by uprooting with an aseptic stainless steel shovel and then shaken to remove loosely attached soil. The tuberous roots with tightly bound soil were added to a 500-ml centrifuge tube with 100 ml of sterile water and vortex-mixed at 200 rpm for 20 min. The soils were collected by centrifugation at 11,000 rpm for 10 min. The RZ was collected as the loosely attached soil on the tuberous roots, and the unplanted field soil was collected as the BK. The washed tuberous roots were used for culturable microorganism analysis. A sample consisted of two plants, and each category was composed of three replications. Twenty-one samples (nine RS, nine RZ, and three BK samples) were stored at low temperatures in ice bags and transported to the laboratory within 8 h. After thoroughly mixed, all samples were stored at -80°C for subsequent DNA extraction.

### Isolation and Identification of Culturable Microorganisms

The culturable fungus was isolated from tuberous roots of *P. heterophylla* with different FW severity using the tissue block method on potato dextrose agar (PDA) medium plates (200-g potato, 20-g D-glucose, 0.5-g MgSO_4_7H_2_O, and 0.5-g KH_2_PO_4_ per liter). All collected tuberous root samples were washed three times in phosphate-buffered saline (PBS) and then sterilized with 75% alcohol for 30 s and sodium hypochlorite for 8 min. We cut the sterilized tuberous roots into 1 × 1 cm pieces, and five pieces were inoculated onto PDA plates for culture at 28°C for 3–5 days. Every sample was repeated by growing on five individual dishes. Single colonies were selected and sub-cultured three times on PDA plates.

Isolation of culturable bacteria from tuberous roots of *P. heterophylla* with different FW severity was conducted using a dilution plate method on Luria-Bertani (LB) agar medium (5-g NaCl, 10-g tryptone, and 5-g yeast extract per liter). Briefly, all collected tuberous root samples were sonicated in 20 ml of PBS solution for 30 min, and an aliquot (2 ml) of the suspension was diluted ten times. An aliquot (100 μl) of the dilution suspension was coated onto LB medium and cultured at 25°C for 2 days, and then, individual colonies were isolated and stored at -80°C in 20% glycerol.

The internal transcribed space (ITS) fragments of the rRNA gene of isolated fungal strains were amplified with universal primers ITS1F (CTTGGTCATTTAGAGGAAGTAA) and ITS2R (GCTGCGTTCTTCATCGATGC), and the whole sequencing of the 16S rRNA genes of isolated bacterial strains was amplified with universal primers 27F (AGAGTTTGATCCTGGCTCAG) and 1492R (TACGGYTACCTTGTTACGACTT). PCR amplicons were verified on a 1% agarose gel. The reaction products were purified using an AxyPrep DNA Gel Extraction Kit (Axygen Biosciences, Union City, CA, United States) and then sequenced on an ABI 3730XL DNA Analyzer (Applied Biosystems, CA, United States) according to the standard protocols by Majorbio Bio-Pharm Technology Co., Ltd. (Shanghai, China). The ITS and 16S sequences were aligned with the NCBI NR database by Nucleotide BLAST^1^ to determine the approximate phylogenetic affiliation of the strains. Taxonomy was confirmed by identity value with a threshold 98% that was selected as the first-ranked of all listed matches.

### DNA Extraction and Internal Transcribed Space and V3V4 Fragment Amplification

According to the manufacturer’s protocols, microbial DNA was extracted from 21 samples using the E.Z.N.A.^§^ soil DNA Kit (Omega Biotech, Norcross, GA, United States). The DNA concentration and purification were determined using a NanoDrop 2000 UV-Vis spectrophotometer (Thermo Scientific, Wilmington, DE, United States). The DNA integrity was confirmed by 1% agarose gel electrophoresis. We amplified the V3V4 hypervariable regions of the bacterial 16S rRNA gene with primers 338F (5′-ACTCCTACGGGAGGCAGCAG-3′) and 806R (5′-GGACTACHVGGGTWTCTAAT-3′) and ITS1 regions of the fungus rRNA gene with primers ITS1F (5′-CTTGGTCATTTAGAGGAAGTAA-3′) and ITS2R (5′-GCTGCGTTCTTCATCGATGC-3′). The PCR reactions were conducted using the following conditions: pre-denaturation for 3 min at 95°C, 27 cycles of denaturation for 30 s at 95°C, annealing for 30 s at 55°C, elongation for 45 s at 72°C, and a final extension at 72°C for 10 min. The PCR reactions were performed in triplicate in a 20 μl of reaction mixture containing 4 μl of 5 × FastPfu Buffer, 2 μl of 2.5 mM dNTPs, 0.8 μl of each primer (5 μM), 0.4 μl of FastPfu Polymerase, and 10 ng of template DNA.

### Illumina MiSeq Sequencing and Analysis

According to the manufacturer’s protocol, the PCR products were isolated and extracted from a 2% agarose gel, purified using the AxyPrep DNA Gel Extraction Kit (Axygen Biosciences, Union City, CA, United States), and then quantified using QuantiFluor-ST (Promega, Madison, WI, United States). Purified amplicons were pooled in equimolar amounts and paired-end sequenced (2 × 300) on an Illumina MiSeq platform (Illumina, San Diego, CA, United States) according to the standard protocols by Majorbio Bio-Pharm Technology Co., Ltd. (Shanghai, China).

After Illumina sequencing, we obtained 0.81-gigabyte (GB) fungi and 0.79-GB bacterial raw sequences deposited in the Sequence Read Archive database at the NCBI (the BioProject accession number: PRJNA803322). Trimmomatic was used to quality-filter and trim the raw sequences, and FLASH was used to merge the paired-end sequences to a tag from the high-quality clean sequences following the criteria as previously described (Yuan et al., 2018). Operational taxonomic units (OTUs) were clustered with a 97% similarity cutoff using UPARSE software (version 7.1^2^). The taxonomy assignment of bacteria was analyzed by the Ribosomal Database Project Classifier algorithm (RDP, V.11.5^3^) against the SILVA rRNA database (SSU123^4^) (Yilmaz et al., 2013) with a 70% confidence threshold. In addition, the taxonomy assignment of fungi was analyzed by the RDP algorithm against the UNITE database (version 6^5^) (Nilsson et al., 2019).

Alpha-diversity indexes evaluated each sample’s richness and species diversity. The observed species, Chao, ACE, Shannon, and Simpson indexes were calculated by the QIIME software (V1.80^6^). Principal coordinate analysis (PCoA) based on the Bray–Curtis distance matrix computed the beta diversity metrics using the “phyloseq” and “vegan” packages of R software (v3.6.2^7^). The phylum composition in different soil groups was calculated by the “statnet” package of R software and was visualized by the “circlize” package of R software. The ternary analysis calculated microbe enrichment by R software’s “vcd” package. The “psych” package calculated the correlation analysis in R software based on the Sparcc method (Yan et al., 2020). The filter conditions were significance level with cutoff value *p* < 0.05 and correlation coefficient with threshold value *r* > 0.6. Finally, the co-occurrence diagrams were visualized by the “igraph” package in R software.

### Growth Assays of Bacterial Isolates From *Pseudostellaria heterophylla* Roots Mixed With Extracts of *Pseudostellaria heterophylla* Tuberous Roots Infected by *Fusarium oxysporum*

A 10-g tuberous root of *P. heterophylla* inoculated with *F. oxysporum* was ground into a powder to prepare an extract. Then, 50 ml of water was added, and the solution was ultrasonicated for 20 min; this solution was used as the control. The suspension was sterile-filtered with a 0.22-μm membrane and then added to LB medium according to the volume ratio of 1:100. Next, 1 μl of culture from 216 bacterial strains isolated from diseased tuberous roots was inoculated in LB medium with aqueous extract and was cultured in a 25°C incubator under the light. Three replicates were carried out for each treatment. Two days after cultivation, we captured images using a Nikon microscope and calculated the growth area of each strain under different treatments using ImageJ software.

### The Pathogen-Induced Attraction Assay

The pathogen-induced attraction assay in the agar medium was carried out as follows. Sodium hypochlorite–sterilized *P. heterophylla* seeds were germinated at 25°C and 80% humidity. Two-week-old plants with the same growth vigor were selected and transferred to the center of the agar medium. They were inoculated with 10 μl of *F. oxysporum* spores (isolated from the tuberous root of *P. heterophylla*, concentration = 5 × 10^5^ CFU/ml); 10 μl of was used as the control. Plants were grown in a light incubator and covered with transparent lids to increase the relative humidity. After 7 days, a sterilized toothpick was placed parallel with the tuberous root at a 0.5-cm distance, and 100 μl of fresh cultures of *Pseudomonas poae* and *Pseudomonas tonnasii* (isolated from the tuberous root of *P. heterophylla*, OD600 = 1) was inoculated on the toothpick. Plants were co-cultured at 25°C and 80% humidity. Images were captured using a Nikon microscope after 7 days of co-cultivation, and the growth distances of *P. poae* and *P. tonnasii* were calculated using ImageJ software. Each treatment was carried out for three replicates.

The pathogen-induced attraction assay in soil was carried out as follows. The preparation of plants and inoculation pathogen were similar to that for the pathogen-induced attraction assay in agar medium. Fresh *P. poae* (10 ml) (OD600 = 1) was sprayed on the soil in pots, and *F. oxysporum*– and ddH_2_O-inoculated plants were cultured in the pots in a light incubator at 25°C and 80% humidity. Seven days after cultivation, images were captured using a Nikon microscope. The numbers of *P. poae* in the tuberous roots were determined by taxon-specific qPCR. In brief, the tuberous root from each treatment was weighed and ultrasonicated for 20 min in PBS. The supernatant was collected and centrifuged for 10 min at 12,000 rpm, and the precipitate was then harvested and used to extract total DNA using an E.Z.N.A.^§^ Soil DNA Kit (Omega Bio-Tek Inc., Norcross, GA, United States). The quality and quantity of each sample’s DNA were examined with a NanoDrop spectrophotometer (Eppendorf, HH, Germany). All qPCRs were carried out in 96-well plates using a Bio-Rad, Hercules, CA, United States CFX Connect Real-Time System (Bio-Rad, Hercules, CA, United States). Taxon-specific primers were used to analyze the *sucD* genes in *P. poae* (Supplementary Table 7). The relative abundance of *P. poae* was calculated from the cycle threshold values and linear regression coefficients derived from the standards of each strain and adjusted to gene copies per gram of tuberous root weight. There were three replicates for each treatment, including six tuberous roots.

### Colonization Assay

In the colonization assay, the preparation of plants was similar to that followed for the disease-induced attraction assay in the agar medium. The plants were co-inoculated with 20 ml of *F. oxysporum* spores (concentration = 5 × 10^5^ CFU/ml) and treated (or not) with 20 ml of *P. poae* suspension (OD600 = 0.5); inoculation with ddH_2_O was used as a control. Plants were cultured under similar conditions as those described above. Fourteen days after cultivation, images were captured using a Nikon microscope, and the relative abundance of *Fusarium* and *Pseudomonas* in tuberous roots was determined by taxon-specific qPCR as described above. Taxon-specific primers for *ITS* genes in *F. oxysporum* are shown in Supplementary Table 7.

### Statistical Analysis

The two-tailed Wilcoxon rank sum test was calculated using the Wilcox.test function in the “stats” package of R. Spearman’s correlation coefficient and significant differences were computed using the corr.test function in the “psych” package of R. Analysis of similarities (ANOSIM) was performed with the anosim function from the “vegan” package of R based on the Bray–Curtis metric method. The Bray–Curtis metric was calculated using the vegdist function from the R package “vegan.”

## Results

### Diversity and Composition of the Microbial Community Were Highly Correlated With the *Fusarium* Wilt Severity in the Rhizosphere and Endosphere of Tuberous Roots

To investigate the microbial composition and diversity of *P. heterophylla* in soils conferring different disease severity, we collected soils with three severities of FW from their authentic production areas in Shibing County (Guizhou Province) (Supplementary Table 1). Accordingly, we collected RS, RZ, and BK samples as described by Shi et al. (2019) and Yuan et al. (2020b; Supplementary Figure 1). The V3V4 and ITS2 regions of the bacterial 16S rDNA and fungal 18S rDNA were amplified by PCR and sequenced on an Illumina MiSeq platform. In total, 1,316,364 high-quality clean V3V4 sequences were obtained with a median sequence per sample value of 62,684 (range 53,296–73,661) from 21 samples. In addition, 1,338,526 high-quality clean ITS2 sequences were obtained with a median sequence per sample value of 63,740 (range 39,024–74,174) from 21 samples (Supplementary Table 1).

The general features of the high-throughput sequencing results of the V3V4 and ITS2 region and taxon numbers at all levels are shown in Supplementary Tables 2, 3, respectively. In total, Good’s coverage for the observed bacterial OTUs and fungal OTUs were 98.8 ± 0.03% and 99.7 ± 0.01% (mean ± SEM), respectively, which resulted in the identification of 4,716 bacterial OTUs and 2,065 fungal OTUs. The number of OTUs in each soil compartment is shown in Supplementary Figure 2. The number of OTUs was no statistical significance for RS or RZ by Kruskal–Wallis rank sum test among the three severities of FW (RS in bacteria, *P* = 0.4911; RZ in bacteria, *P* = 0.2881; RS in fungi, *P* = 0.8752; RZ in fungi, *P* = 0.9565). Alpha-diversity analysis showed that Ace, Chao, and Sobs were significant differences in the bacterial community between RZH and RZN but not in the fungal community (Duncan’s multiple range test, *P* < 0.05) (Supplementary Tables 4, 5).

**FIGURE 1:**
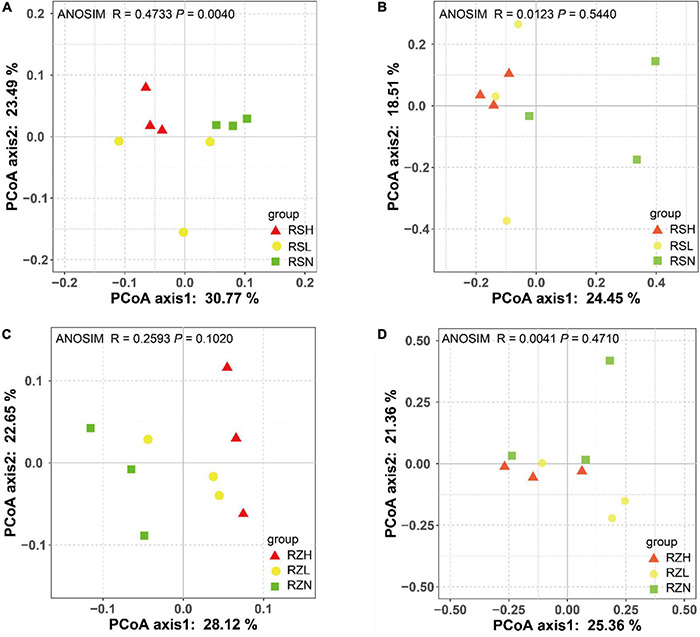
*Fusarium* wilt separated the bacterial community in **(A)** RS but not that in **(C)** RZ, and it did not divide the fungal community in **(B)** RS or **(D)** RZ. PCoA was used to visualize the dissimilarity of bacterial and fungi compositions at the OTU level based on the Bray–Curtis metric. Red triangles, yellow circles, and green squares denote samples in group H (RSH and RZH), L (RSL and RZL), and N (RSN and RZN), respectively.

Principal coordinate analysis (beta diversity) was performed to visualize the similarity and dissimilarity in the bacterial and fungal OTUs obtained from 16S rRNA and 18S rRNA gene amplicon sequencing using the Bray–Curtis metric. The results revealed that RS separated the bacterial community according to the severity of FW (H, triangle; L, circular; and N, square). ANOSIM analysis also showed that there were significant differences in the beta diversity of the bacterial community in RS (*R* = 0.4733, *P* = 0.0040) but not in RZ (*R* = 0.2593, *P* = 0.1020) (Figure 1). However, there was no significant difference in the beta diversity of the fungal community among the severities of FW in RZ (ANOSIM, *R* = 0.0041, *P* = 0.4710) and RS (ANOSIM, *R* = 0.0123, *P* = 0.5440). This finding indicated that the presence of the pathogen altered the composition of the bacterial community in RS.

**FIGURE 2:**
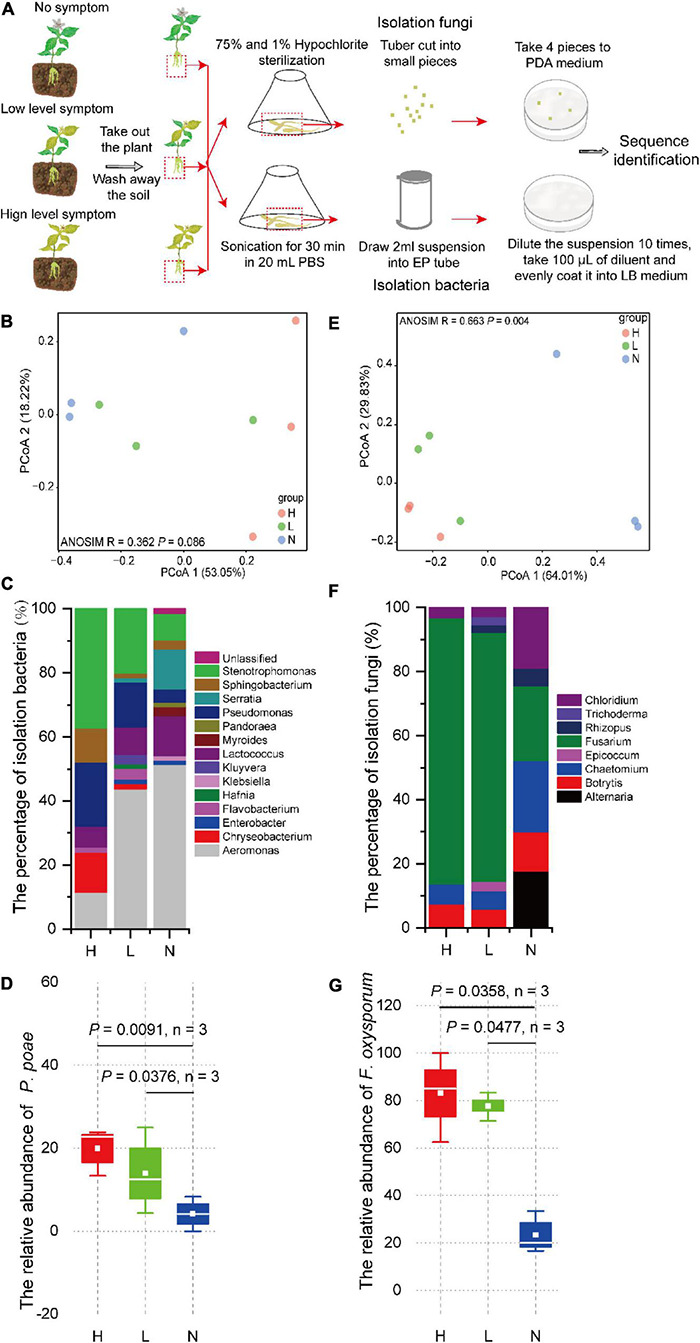
**(A)** Culturable microorganisms isolated and identified from tuberous roots of *P. heterophylla* with different FW severity. The differences in **(B)** bacterial and **(E)** fungal community compositions among high, low, and no FW severity levels. PCoA was used to visualize the dissimilarity of bacterial and fungal compositions based on the Bray–Curtis metric at the genera level. Red, green, and blue circles denote samples in groups H, L, and N, respectively. The composition of culturable **(C)** bacteria and **(F)** fungal communities among high, low, and no FW severity levels. The relative abundance of **(D)** *P. poae* and **(G)** *F. oxysporum* was obviously and positively associated with FW severity. The two-tailed Wilcoxon test evaluated the significant differences.

To confirm whether the diversity and composition of the microbial community are related to disease severity, we investigated culturable fungi and bacteria on the tuberous root of *P. heterophylla* with different severity of FW. Ninety-four strains of fungi and 216 strains of bacteria were isolated, and then, the V3V4 and ITS2 genes were amplified and sequenced. PCoA was performed based on the V3V4 and ITS2 rDNA gene amplicon sequences of bacteria and fungi using the Bray–Curtis metric to visualize the similarity and dissimilarity in bacterial and fungal communities among soil sample metrics. The results revealed that the severities of FW separated the bacterial and fungal communities in the tuberous root (H, red circular; L, green circular; and N, blue circular). ANOSIM analysis also showed slight differences in the bacterial community (*R* = 0.362, *P* = 0.086), and there were significant differences in the H, L, and N tuberous root in the fungal community (Figures 2B,E). This finding indicated that the composition of the bacterial community was closely associated with FW within the tuberous roots.

### The Abundance of *Fusarium* Wilt Pathogen *Fusarium oxysporum* and Beneficial Bacterial *Pseudomonas poae* Was Significantly Correlated With *Fusarium* Wilt Severity

**FIGURE 3:**
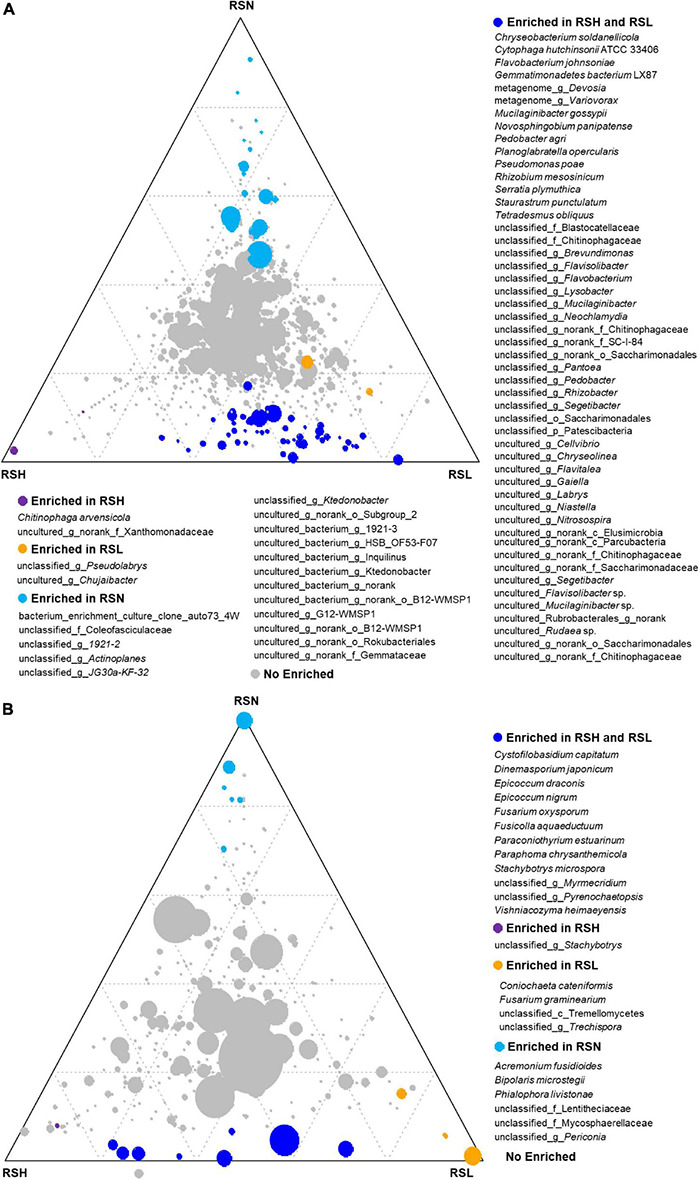
The difference of (*P* < 0.05) **(A)** bacterial and **(B)** fungal species among RSH, RSL, and RSN evaluated using the two-tailed Wilcoxon test. For each species, blue, red, yellow, and gray circles represent the differential genera enriched in RSH and RSL, RSH, RSL, and RSN, respectively.

The ternary phase diagram was applied to identify the differential taxa of the bacterial community among H, L, and N in the RS compartment (Figure 3A). At the species level, 71 bacterial species were found to be significantly (*P* < 0.05) different among RSH, RSL, and RSN, of which 50 species were enriched in both RSH and RSL, and 2, 2, and 17 were only enriched in RSH, RSL, and RSN, respectively. Among them, *Chryseobacterium soldanellicola*, *Cytophaga hutchinsoni*, *Flavobacterium johnsoniae*, *Gemmatimonadetes bacterium* LX87, *Mucilaginibacter gossypii*, *Novosphingobium panipatense*, *Pedobacter agri*, *Planoglabratella opercularis*, *P. poae*, *Rhizobium mesosinicum*, *Serratia plymuthica*, *Staurastrum punctulatum*, and *Tetradesmus obliquus* were significantly enriched in both RSH and RSL (one-way ANOVA; *P* < 0.05). In addition, the analysis of the composition of culturable bacteria indicated that the relative abundance of *P. poae* was enriched in both RSH (*P* = 0.0091) and RSL (*P* = 0.0376) (Figures 2C,D). Many previous studies showed that *P. poae* (Cho et al., 2007; Zachow et al., 2015; Xia et al., 2019; Ren et al., 2021) and *M. gossypii* (Madhaiyan et al., 2010) participate in plant growth-promoting activity and exhibit biocontrol potential to suppress disease invasion. Our results showed that the plant-beneficial microorganism consortium (i.e., *P. poae* and *M. gossypii*) was significantly enriched and correlated with the severities of FW.

The ternary analysis was applied to identify the differential taxa of the fungal community among H, L, and N in the RS compartment (Figure 3B). At the species level, 23 fungal species were significantly (*P* < 0.05) different among RSH, RSL, and RSN, of which 12 species were enriched in both RSH and RSL, and 1, 4, and 6 were only enriched in RSN, RSL, and RSN, respectively. *Cystofilobasidium capitatum*, *Dinemasporium japonicum*, *Epicoccum draconis*, *E. nigrum*, *F. oxysporum*, *Fusicolla aquaeductuum*, *Paraconiothyrium estuarinum*, *Paraphoma chrysanthemicola*, *Stachybotrys microspora*, and *Vishniacozyma heimaeyensis* were significantly enriched in both RSH and RSL (one-way ANOVA; *P* < 0.05). *Coniochaeta cateniformis* and *F. graminearium* were also considerably enriched in RSL (one-way ANOVA; *P* < 0.05). The analysis of the composition of culturable fungi indicated that the relative abundance of *F. oxysporum* was enriched in both RSH (*P* = 0.0358) and RSL (*P* = 0.0477) (Figures 2F,G). The results showed that the FW pathogen *F. graminearium* was significantly enriched and correlated with the severities of FW.

### Correlation Analysis Showed That *Pseudomonas poae* Might Be Regulated by *Fusarium oxysporum*

**FIGURE 4:**
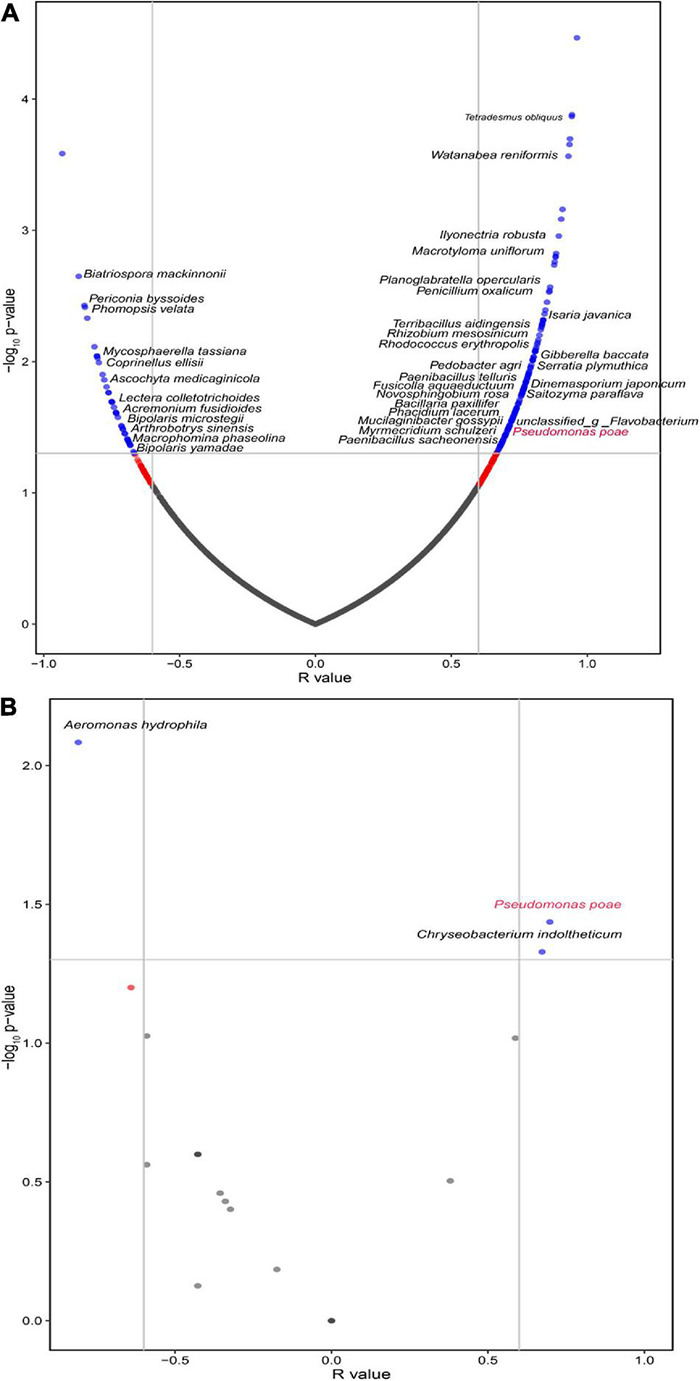
The volcano plot for correlations between *F. oxysporum* and the microbial community in the **(A)** rhizosphere and **(B)** endosphere of the tuberous roots. The green points represent significantly correlated species (Sparcc, *r* > 0.6, *P* < 0.05).

The interaction between different microorganisms is one of the most important driving factors of population structure and dynamics because they can coexist, attract, or repel each other (Faust et al., 2012; Falony et al., 2016). Hence, the Spearman correlation analysis showed that *F. oxysporum* was positively correlated with 135 species and negatively associated with 39 species in the rhizosphere of tuberous roots (Supplementary File 2). Intriguingly, we found that *F. oxysporum* was significantly and positively correlated with *P. poae* and *M. gossyppii* (Figure 4A). In addition, we isolated the culturable microorganisms from tuberous roots with different FW severity and found that *F. oxysporum* was significantly and positively correlated with *P. poae* within the tuberous roots (Figure 4B). Many reports have shown that *Pseudomonas* could be attracted to roots depending on plant disease outbreak (Cazorla et al., 2006; Kamilova et al., 2008; Zhuang et al., 2020). These results showed that *P. poae* might be regulated by *F. oxysporum* in the *P. heterophylla* tuberous root.

### The Extracts From the Tuberous Roots of *Pseudostellaria heterophylla* Inoculated With *Fusarium oxysporum* Promoted the Growth of *Pseudomonas poae*

**FIGURE 5:**
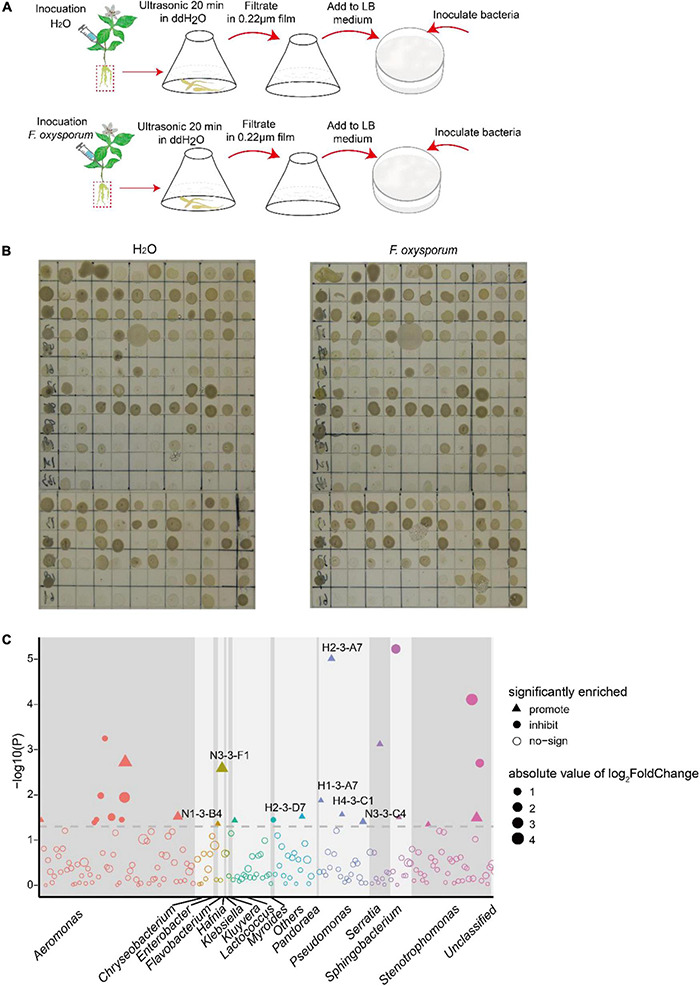
The extracts of the tuberous root of *P. heterophylla* inoculated with *F. oxysporum* affect the growth of isolates. **(A)** We ground the tuberous roots of *P. heterophylla* infected with *P. oxysporum* into powder, added 50 ml of water, and ultrasonicated the solution for 20 min; inoculation with H_2_O served as a control. The suspension was sterilized with a 0.22-μm membrane and then added to LB medium according to the volume ratio of 1:100. Next, 1 μl of cultures of 216 bacterial strains isolated from diseased tuberous roots were inoculated in LB medium with aqueous extract and cultured in an incubator at 25°C with light. After 2 days of cultivation, compared with the control, the **(B)** growth phenotype diagram and **(C)** the Manhattan map show the effects on isolates in aqueous extracts. The dashed line represents the logarithm of *P* = 0.05, and triangles and circles represent the effects of promotion and inhibition, respectively. Different colors and sizes represent different taxa of isolates and the absolute value of log_2_FC.

The growth assay evaluated the effect on isolates of the crude extract from the tuberous root of *P. heterophylla* inoculated with *F. oxysporum*. Fifteen and 11 bacterial isolates were significantly promoted and inhibited by the aqueous extract, respectively. Notably, the growth of *P. poae* (H1-3-A7, H2-3-B7, H4-3-C1, and N3-3-C4) was particularly (two-tailed Wilcoxon test, *P* < 0.05) promoted by the aqueous crude extract of tuberous roots inoculated with *F. oxysporum* (Figure 5 and Supplementary Table 6). These results indicated that the extract from the tuberous root of *P. heterophylla* inoculated with *F. oxysporum* might attract *P. poae* and promote their growth.

### *Pseudostellaria heterophylla* Indirectly Assembled *Pseudomonas poae* in Its Rhizosphere and Endosphere Upon *Fusarium oxysporum* Infection

To further investigate if *Pseudomonas* can be attracted to colonize within the tuberous root, greenhouse colonization assay and qPCR were used to quantify its colonization difference *in vivo*. We designed a high-stress colonization simulation experiment by treating the soil with H_2_O (as a control), *Pseudomonas*, *Fusarium*, or a mixture of *Pseudomonas* and *Fusarium* (Supplementary Figure 3). At 14 days after injection, the abundance of *P. poae* in the tuberous root was detected *via* the copies of *sucD* quantified by qPCR using taxon-specific primers (Supplementary Table 7). Our results showed that the gene copies of *sucD* in *P. poae* treatment (up to 3.29 × 10^10^) were significantly (two-tailed Wilcoxon test, *P* = 0.0043, *n* = 12) higher than those in the H_2_O treatment (0.88 × 10^10^).

**FIGURE 6:**
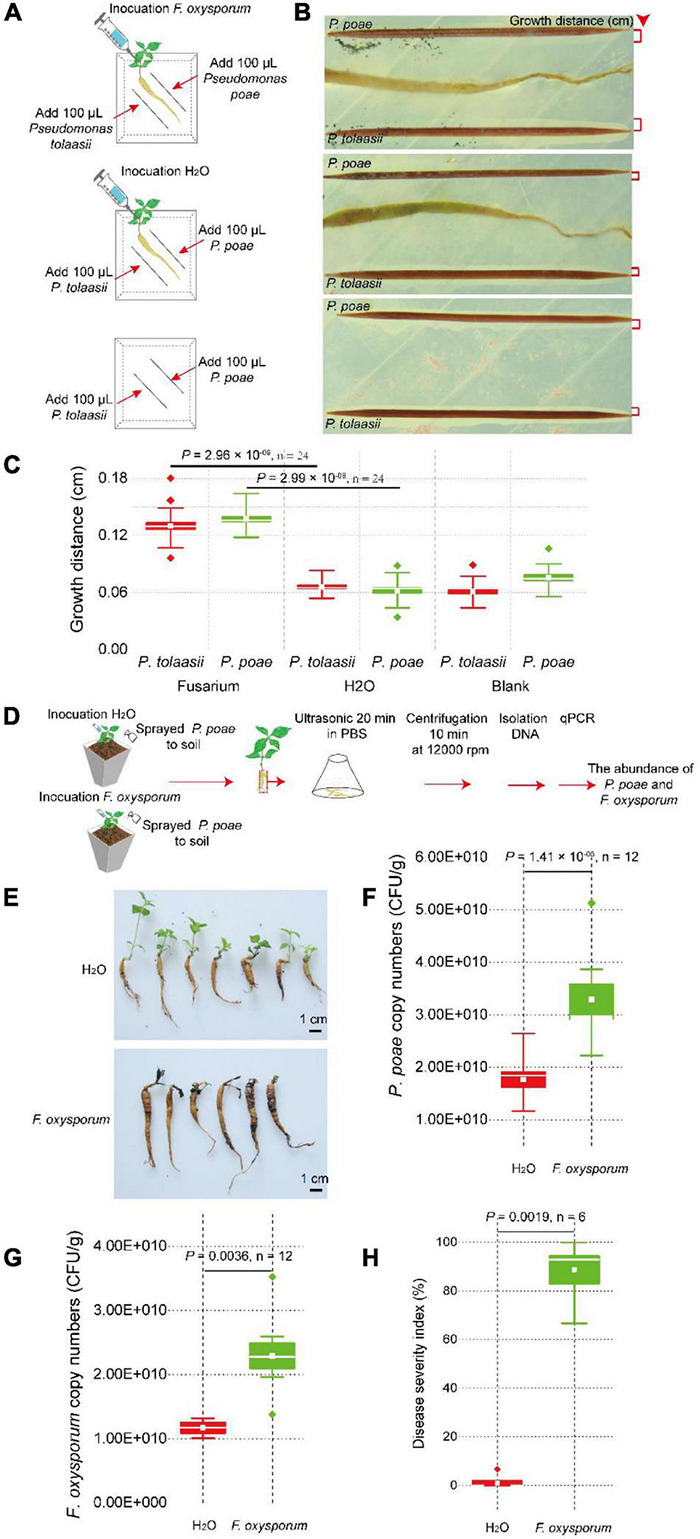
*Pseudostellaria heterophylla* inoculated with *F. oxysporum* attracted *P. poae and P. tonnasii* in agar medium. **(A)** Two-week-old plants with the same growth vigor were selected and transferred to the center of the agar medium. They were inoculated with 10 μl of *F. oxysporum* spores (concentration = 5 × 10^5^ CFU/ml) or 10 μl of ddH_2_O. After 7 days, a sterilized toothpick was placed parallel to the tuberous root at a 1-cm distance, and 100 μl of new cultures of strains *Pseudomonas poae* or *P. tonnasii* (OD600 = 1) were inoculated on the toothpick. The growth distance of *Pseudomonas* was calculated by ImageJ software, as shown in **(B,C)**. **(D)** *P. heterophylla* inoculated with *F. oxysporum* attracted the *P. poae* in the soil. **(E)** The FW severity in plants treated with or without *F. oxysporum*. **(F)** The density of *P. poae* in tuberous roots inoculated with *F. oxysporum* or H_2_O. **(G)** Changes in the *F. oxysporum* abundance in tuberous roots inoculated with *F. oxysporum* or H_2_O. **(H)** Changes in the disease severity index (DSI) of *P. heterophylla* with or without inoculated *F. oxysporum*. The two-tailed Wilcoxon test evaluated the significant differences.

Notably, the gene copies of *sucD* in the *P. poae* and *F. oxysporum* treatment (up to 6.57 × 10^10^) group was significant (two-tailed Wilcoxon test, *P* = 0.0079, *n* = 12) higher than those in the *P. poae* treatment group (3.29 × 10^10^). Moreover, the root populations of *F. oxysporum* were analyzed *via* the gene copies of *ITS* quantified by qPCR using taxon-specific primers (Supplementary Table 7). The copies of *ITS* in the *F. oxysporum* treatment group (up to 9.70 × 10^10^) were significantly (two-tailed Wilcoxon test, *P* = 0.0286, *n* = 12) higher than those in the H_2_O treatment group (2.27 × 10^10^). Interestingly, the copies of *sucD* in the *P. poae* and *F. oxysporum* treatment groups (up to 3.64 × 10^10^) were significant (two-tailed Wilcoxon test, *P* = 0.0286, *n* = 12) lower than those in the *F. oxysporum* treatment group (9.70 × 10^10^). The DSI in the *P. poae* and *F. oxysporum* treatment group was significant (two-tailed Wilcoxon test, *P* = 0.0080, *n* = 12) lower than that in the *F. oxysporum* treatment group. Together, these results further showed that *F. oxysporum* promoted *P. poae* to colonize within the tuberous root. In contrast, *P. poae* inhibited *F. oxysporum* from infecting its host (Figure 6H and Supplementary Figure 3E).

The pathogen-induced attraction assay *via* physical isolation was used to investigate whether *F. oxysporum* directly or indirectly induces *P. poae* to colonize on the surface of the tuberous root. The pathogen-induced attraction assay in the agar medium, *F. oxysporum* or H_2_O, was inoculated on *P. heterophylla* seedlings, and *P. poae* and *P. tonnasii* were parallel to the tuberous root and inoculated on a medium at a distance of 0.5 cm (Figure 6A). At 7 days after co-cultivation, the growth distance of *Pseudomonas* was calculated by ImageJ software. There was no significant difference in the growth distance of *P. poae* and *P. tonnasii* between the H_2_O and blank (two-tailed Wilcoxon test, *P* > 0.05, *n* = 24). Compared to the H_2_O treatment, the growth distances of *P. poae* (two-tailed Wilcoxon test, *P* = 2.96 × 10^–09^, *n* = 24) and *P. tonnasii* (two-tailed Wilcoxon test, *P* = 2.99 × 10^–09^, n = 24) in the *Fusarium* treatment group were significantly high (Figures 6B,C). In addition, in the greenhouse assay (Figure 6D), we found that the relative copies of *sucD* of *P. poae* in the *F. oxysporum* treatment were significant (two-tailed Wilcoxon test, *P* = 1.43 × 10^–05^, *n* = 12) higher than in the H_2_O treatment (Figure 6F). Furthermore, the number of *F. oxysporum* in the *F. oxysporum* treatment group was significantly (two-tailed Wilcoxon test, *P* = 0.0036, *n* = 12) higher than that in the H_2_O treatment group (Figure 6G). The DSI in the *F. oxysporum* treatment group was significantly (two-tailed Wilcoxon test, *P* = 0.0019, *n* = 6) higher than in the H_2_O treatment group (Figures 6E,H). Together, these results further showed that pathogen *F. oxysporum* could mediate *P. heterophylla* to assemble *P. poae*.

## Discussion

### The High Population of Host-Specific Pathogens and High Colonization Ability in the Rhizosphere and Endosphere Determine the *Fusarium* Wilt Incidence

The microorganisms in soil and root are essential for plant health. The high enrichment of pathogens in rhizosphere soil is the main factor responsible for plant disease occurrence (Berendsen et al., 2012; Zhao et al., 2016; Tan et al., 2017; Li et al., 2019; Liu et al., 2019a; Luo et al., 2019; Wu et al., 2019). Our results showed that the high abundance of *F. oxysporum* in continuous monoculture soil is the main factor leading to the high occurrence of FW in *P. heterophylla* (Figure 3B). This result is consistent with previous studies that determined that continuous monoculture enriches pathogens and disturbs the structure of microbial communities, leading to a high incidence of FW (Wu et al., 2016a; Zhao et al., 2016; Yuan et al., 2020a). In addition, we found that the populations of *F. oxysporum* in tuberous roots were positively correlated with the severity of FW, which showed that the high ability of the pathogen to colonize the plant is a direct factor in the severity of FW (Figure 2). Many studies have demonstrated that an outbreak of the disease can be initiated only when the pathogen reaches a certain threshold and successfully colonizes itself in the plant (Bakker et al., 2013). Our results showed that the high populations of *F. oxysporum* in rhizosphere soil and their ability to colonize tuberous roots determine the occurrence of FW.

### Pseudomonas Is Widely Mediated by a Variety of Plant Pathogens to Enrich and Colonize in Rhizosphere Soil and Within Roots to Resist Diseases

Plants widely regulate Pseudomonas in response to biotic and abiotic stress. Our study found that pathogen-mediated *P. heterophylla* enriched Pseudomonas in rhizospheric soil and participated in decreasing the incidence of FW. This result is consistent with studies conducted with maize (Zhu et al., 2021), barley (Schreiner et al., 2010; Dudenhffer et al., 2016), wheat (Landa et al., 2002), pea (Landa et al., 2002), carnations (Lemanceau et al., 1992), and *Panax notoginseng* (Zhang et al., 2020b). In addition, Pseudomonas was also mediated by pathogens such as *Setosphaeria turcica*, *Gaeumannomyces gramini*, and *Fusarium* spp. (Lemanceau et al., 1992; Landa et al., 2002; Zhang et al., 2020b; Zhu et al., 2021). Furthermore, Pseudomonas was induced by *F. oxysporum* to colonize on the tuberous roots, which was similar to the results of a previous study showing that leaf pathogens can mediate the colonization of Pseudomonas on Arabidopsis roots (Léon-Kloosterziel et al., 2005). Several members of Pseudomonas can produce jasmonic acid, salicylic acid, and ethylene to promote plant growth (Léon-Kloosterziel et al., 2005), induce systemic resistance (Verhagen et al., 2010), and directly produce volatile substances, lipopeptide (Zachow et al., 2015), and 2,4-diacetylphloroglucinol (Keel et al., 1992; Schouten et al., 2004) to inhibit pathogens. These results indicate that pathogens widely regulate Pseudomonas in the rhizosphere and within roots, and plants recruit it to confront pathogen invasion.

### Pathogen-Mediated *Pseudostellaria heterophylla* Assembled a Beneficial Bacterial Consortium to Increase Its Resistance to *Fusarium* Wilt

We found that *P. heterophylla* enhanced the regulation of the bacterial community depending on the severity of FW (Supplementary Tables 8, 9). The ternary phase showed that *P. heterophylla* strongly regulated many beneficial microbes such as *Cytophaga hutchinsoni*, *Mucilaginibacter gossypii*, *Novosphingobium panipatense*, *Rhizobium mesosinicum*, and *P. poae* to enrich them in rhizosphere soil and colonize them within tuberous roots because these species possess multiple helpful functions to maintain plant health. *N. panipatense* can enhance tolerance in plants to multiple heavy metals (Chettri and Singh, 2019). *C. hutchinsoni* is essential for ion assimilation (Gao et al., 2020), and *M. gossypii* produces large amounts of extracellular polysaccharides and possesses plant-growth-promoting traits (Madhaiyan et al., 2010). *R. mesosinicum* can fix nitrogen and increase the nitrogen source of plants (Lin et al., 2009; Xu et al., 2015). The endophytic *P. poae* produces the lipopeptide poaeamide, which is involved in pathogen suppression and root colonization (Zachow et al., 2015; Xia et al., 2019; Ren et al., 2021). Together, these results show that, when *P. heterophylla* is infected by a pathogen, it can regulate and attract multiple functional microbe consortia that can promote nutrient acquisition, reduce heavy metal toxicity, and produce antibiotics to alleviate diseases.

### The Phenomenon of the Plant Recruiting Beneficial Bacteria Upon Pathogen Infection Can Be Used to Develop New Biocontrol Technology

Biological control has become an important ecological and sustainable agricultural method for plant disease control (Huang et al., 2017; Zhang et al., 2020a). In our study, *P. poae* directly inhibited the growth of *F. oxysporum* and reduced its pathogenicity. Colonization assay also showed that *P. poae* could decrease the ability of *F. oxysporum* to colonize tuberous roots, thus decreasing the incidence of FW (Figure 5 and Supplementary Figures 3B,E). These results suggest that *P. poae* may act as a potential biocontrol agent to ward off FW, which is consistent with previous studies showing that *P. poae* exhibits plant growth-promoting activity and biocontrol potential to suppress disease invasion (Cho et al., 2007; Zachow et al., 2015; Xia et al., 2019; Ren et al., 2021). It is public knowledge that biocontrol agents with low effective colonization in plants due to their difficult long-lasting survival are significantly limited in their application. Therefore, we can take advantage of the phenomenon that pathogens induce plants to enrich their beneficial bacteria consortia to develop a biological control technology in the future. We hypothesize that this technique can be modified to use dead pathogens to stimulate long-lasting defense actions and subsequently improve the biocontrol agent’s colonization ability and survival ability to enhance its control effect.

Previously, many studies showed that plants regulate the soil microbes *via* root exudates. In our research, the extracts of *P. heterophylla* tuberous roots infected by *F. oxysporum* conferred significant changes that significantly promoted the growth of *P. poae* (Figure 5). This result suggests that *F. oxysporum* infection mediated the *P. heterophylla* tuberous root to produce some metabolites promoting the growth of *P. poae*. Previous studies with wheat plants support this finding, because after pathogen infection, wheat can secrete the metabolites pyoluteorin and 2,4-diacetylphloroglucinol to enrich and colonize *Pseudomonas* in rhizosphere soil and tuberous roots to inhibit the occurrence of diseases (Keel et al., 1992; Lemanceau et al., 1992; Maurhofer et al., 1995; Landa et al., 2002; Weller et al., 2002).

Similarly, Arabidopsis plants infected by bacterial pathogens secreted elevated levels of malic acid. In a dose-dependent manner, malic acid stimulated *Bacillus subtilis* FB17 binding to and biofilm formation on the roots (Rudrappa et al., 2008, 2010). In addition, Zhang et al. showed that *P. heterophylla* tuberous root extracts could promote the growth of *Bacillus thuringiensis* and *Serratia marcescens* (Zhang et al., 2016). These results demonstrated that *P. heterophylla* infected with *F. oxysporum* can adjust its microbiome and may specifically recruit *P. poae* to colonize on tuberous roots *via* secreting uncertain metabolites. A greater understanding of the plant metabiotic basis of disease-induced recruitment of beneficial root-associated microbes could unlock new possibilities for plant disease management technology that more effectively drives the beneficial microbes against pathogens, with enhanced capacities for controlling the disease.

## Conclusion

We present the schematic of how FW-mediated *P. heterophylla* regulates the microbes in the soil and tuberous roots under a continuous monocropping regime (Supplementary Figure 5). Our data suggest that pathogen-mediated *P. heterophylla* promoted and assembled plant-beneficial microbes against plant disease. Therefore, deciphering the beneficial associations between pathogen-mediated *P. heterophylla* and microbes can provide novel insights into the implementation and design of disease management strategies. However, the hypothetical model remains incomplete and exudates of *P. heterophylla* stimulated by *Fusarium* that attracts *Pseudomonas* need to be discovered. Furthermore, additional studies verifying its functions need to be implemented in the future.

## Data Availability Statement

The datasets presented in this study can be found in online repositories. The names of the repository/repositories and accession number(s) can be found below: NCBI (accession: PRJNA803322).

## Author Contributions

TZ and LH conceived and supervised the project. Q-SY, LW, and HW designed the experiment. Q-SY, XW, and XO collected the samples. Q-SY, YY, and JX performed parts of the experiment. YG and WJ analyzed the data. TZ, LH, LG, and XC edited the manuscript. All authors read and approved the final version of the manuscript.

## Conflict of Interest

The authors declare that the research was conducted in the absence of any commercial or financial relationships that could be construed as a potential conflict of interest.

## Publisher’s Note

All claims expressed in this article are solely those of the authors and do not necessarily represent those of their affiliated organizations, or those of the publisher, the editors and the reviewers. Any product that may be evaluated in this article, or claim that may be made by its manufacturer, is not guaranteed or endorsed by the publisher.

## Footnotes

1
https://blast.ncbi.nlm.nih.gov/Blast.cgi

2
http://drive5.com/uparse/

3
http://rdp.cme.msu.edu/

4
https://www.arb-silva.de/

5
https://unite.ut.ee/

6
http://qiime.org/

7
https://www.r-project.org/
