# Supplementary material for: Pathogen-Mediated Assembly of Plant-Beneficial Bacteria to Alleviate Fusarium Wilt in Pseudostellaria heterophylla
Source: Front Microbiol. 2022 Mar 30;13:842372. doi: 10.3389/fmicb.2022.842372 (PMC9005978; doi:10.3389/fmicb.2022.842372)
Supplement: Supplementary file 2 [file Table_1.docx]

**Pathogen-mediated assembly of plant-beneficial bacteria to alleviate *Fusarium* wilt in *Pseudostellaria heterophylla***

Qing-Song Yuan^1^**^#^**, Lu Wang^1^**^#^**, Hui Wang^1^, Xiaoai Wang^1^, Weike Jiang^1^, Xiaohong Ou^1^, Chenghong Xiao^1^, Yanping Gao^1^, Jiao Xu^1^, Ye Yang^3^, Xiuming Cui^3^, Qin Han^4^, Lanping Guo^2^**^*^**, Luqi Huang^2^**^*^**, and Tao Zhou^1^**^*^**

^1^ Guizhou University of Traditional Chinese Medicine, Guiyang 550025, Guizhou, China; [yqs198609031006@126.com](mailto:yqs198609031006@126.com) (QSY); 1076522543@qq.com (LW); [1785227180@qq.com](mailto:1785227180@qq.com) (HW); 1925426266@qq.com (XW); [jwk_88@163.com](mailto:jwk_88@163.com) (WJ); [ogh1986@163.com](mailto:ogh1986@163.com) (XO); [xiaochenghong1986@126.com](mailto:xiaochenghong1986@126.com) (CX); gaoyanping087@gzy.edu.cn (YG); [xujiao2008mzk@163.com](mailto:xujiao2008mzk@163.com) (JX); [taozhou88@163.com](mailto:taozhou88@163.com) (TZ)

^2^ National Resource Center for Chinese Materia Medica, China Academy of Chinese Medical Sciences, Beijing 100700, China; [glp01@126.com](mailto:glp01@126.com) (LG); [huangluqi01@126.com](mailto:huangluqi01@126.com) (LH);

^3^ Faculty of Life Science and Technology, Kunming University of Science and Technology, Kunming 650050, Yunnan, China; [yangyekm@163.com](mailto:yangyekm@163.com) (YY); s[anqi37@vip.sina.com](mailto:anqi37@vip.sina.com) (XC)

^4^ College of Plant Science and Technology, Huazhong Agricultural University, Wuhan 430070, Hubei, China; [hanqin@mail.hzau.edu.cn](mailto:hanqin@mail.hzau.edu.cn) (QH)

**^#^**Authors have equal work in this paper

**Corresponding Authors**

**^*^**Lanping Guo, PhD

National Resource Center for Chinese Materia Medica, China Academy of Chinese Medical Sciences, Beijing 100700, China

[glp01@126.com](mailto:glp01@126.com)

*Luqi Huang, PhD

National Resource Center for Chinese Materia Medica, China Academy of Chinese Medical Sciences, Beijing 100700, China

huanglupi01@126.com

**^*^**Tao Zhou, PhD

Guizhou University of Traditional Chinese Medicine, Dongqingnan Road, Guiyang 540025, China

[taozhou88@163.com](mailto:taozhou88@163.com)


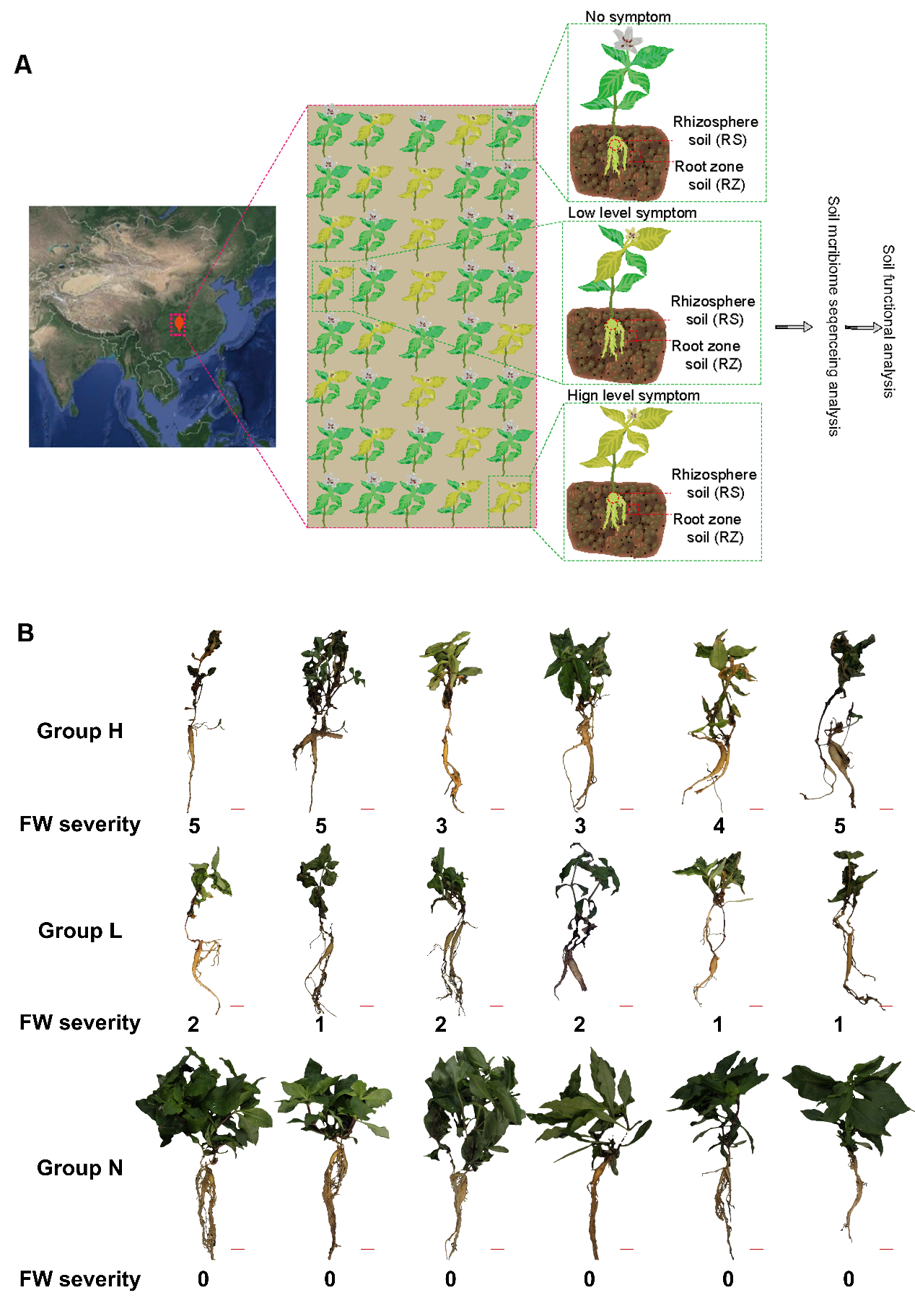


Summary of sampling in this study. The sampling field (27°4'21" N, 108°8'0" E, and 759 m a.s.l.) was located in Shibing County in Guizhou Province. Eighteen Taizishen plants were selected and divided into three groups (H, the group with high FW severity levels; L, the group with low FW severity levels; N, the group with no syndrome of FW) according to wilt severity. Two soil-root system compartments (rhizosphere soil (RS) and root zone soil (ZS) were collected and analyzed for each plant.


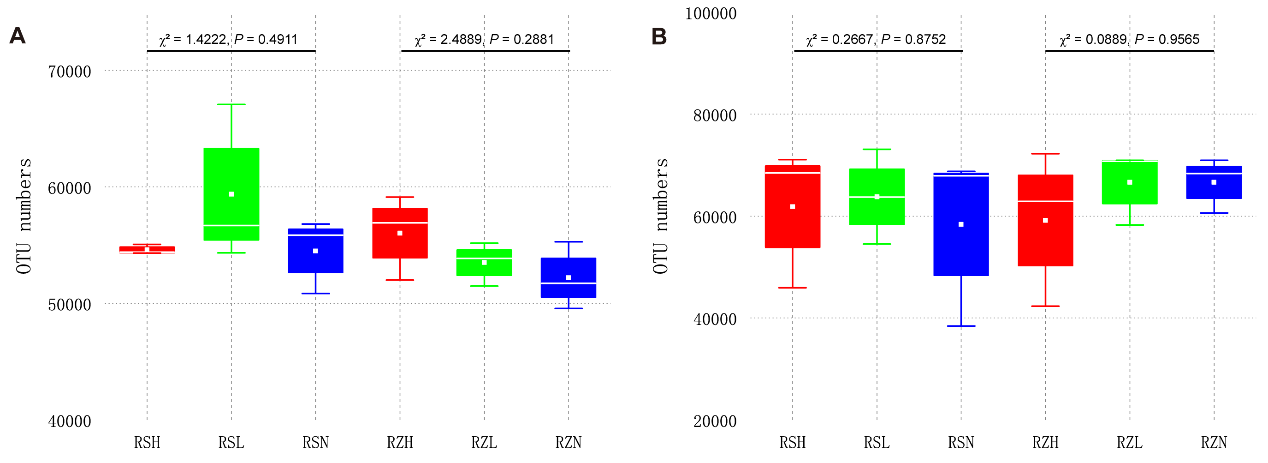


**Fig. S2** No differences among high, low, and no syndrome *Fusarium* wilt severity levels were observed in the bacterial community (**A**) and fungal community (**B**) of both RS and RZ.


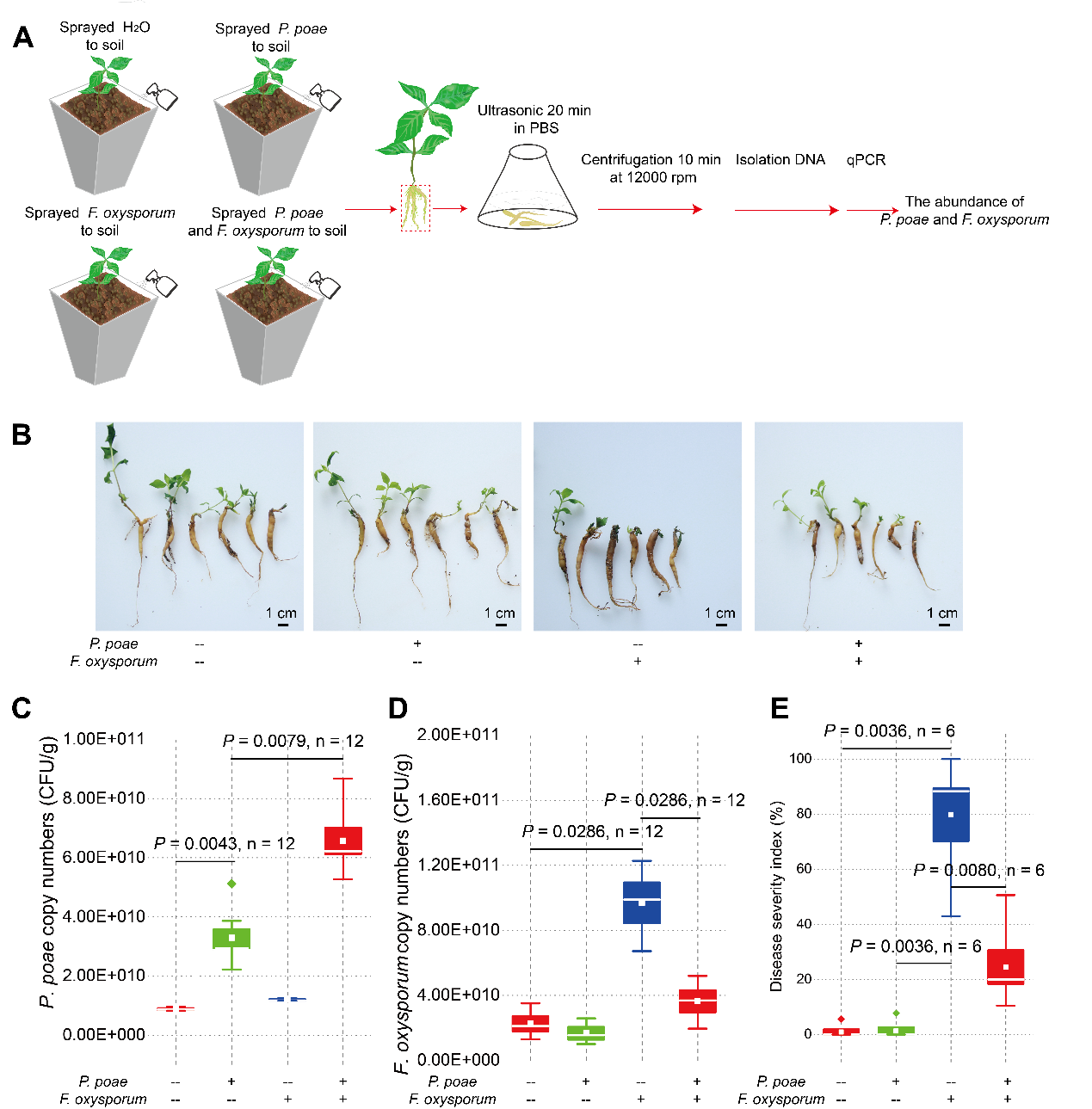


**Fig. S3** *Fusarium* and *Pseudomonas* influence each other on their colonization. The plants were cultured in soil spraying with 20 mL *F. oxysporum* spores (concentration = 5x10^5^ CFU/mL) and the *P. poae* suspension (OD600=0.5), or *F. oxysporum* spores (concentration = 5x10^5^ CFU/mL) or the *P. poae* suspension (OD600=0.5), or ddH_2_O (**A**). Fourteen days after cultivation, we calculated the FW severity of Taizishen treated with or without *F. oxysporum* or/and *P. poae* (**B**). The changes of the *P. poae* density in tuberous root with or without *F. oxysporum* or/and *P. poae* (**C**). The changes of the *F. oxysporum* abundance in tuberous root with or without *F. oxysporum* or/and *P. poae* (**D**). The changes in the disease severity index (DSI) of Taizishen with or without *F. oxysporum* or/and *P. poae* (**E**). The two-tailed Wilcoxon test evaluated the significant differences.


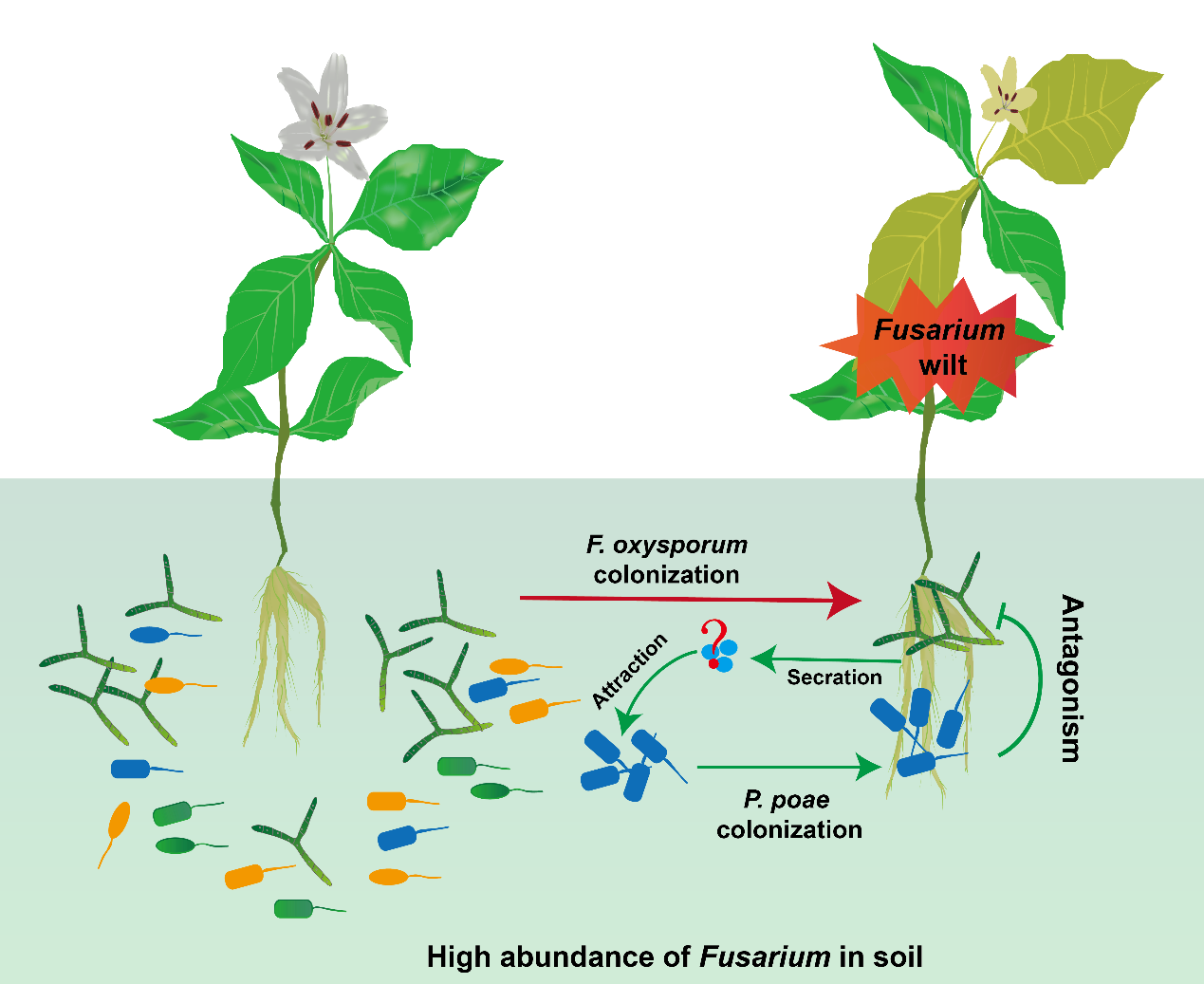


**Fig. S4** It is the hypothetical model that the soil microorganisms influence the incidence of FW and the intelligence of *P. heterophylla* against it in a continuous monoculture field.

**Table S1** The sequence number of 16S rRNA and 18S rRNA in all soil samples

| **Compartment** | **Disease grade** | **V3V4 Seq_num** | **ITS Seq_num** | **Sample ID** |
| --- | --- | --- | --- | --- |
| Bulk soil | - | 64895 | 65653 | BK |
| Bulk soil | - | 62260 | 59084 | BK |
| Bulk soil | - | 57272 | 68873 | BK |
| Rhizosphere | N | 56140 | 69204 | RSN |
| Rhizosphere | N | 63772 | 39024 | RSN |
| Rhizosphere | N | 62699 | 68721 | RSN |
| Rhizosphere | L | 65439 | 55656 | RSL |
| Rhizosphere | L | 64397 | 74174 | RSL |
| Rhizosphere | L | 73661 | 64221 | RSL |
| Rhizosphere | H | 64611 | 71777 | RSH |
| Rhizosphere | H | 65142 | 46397 | RSH |
| Rhizosphere | H | 65623 | 70108 | RSH |
| Root zoom | N | 53296 | 61699 | RZN |
| Root zoom | N | 60037 | 71965 | RZN |
| Root zoom | N | 54975 | 69067 | RZN |
| Root zoom | L | 62414 | 59105 | RZL |
| Root zoom | L | 63525 | 71714 | RZL |
| Root zoom | L | 63653 | 72524 | RZL |
| Root zoom | H | 58255 | 73055 | RZH |
| Root zoom | H | 65564 | 63760 | RZH |
| Root zoom | H | 68684 | 42745 | RZH |

**Table S2** General features of the bacterial community sequencing results in soil samples

| **Sample**  **ID** | **Number of sequences** | **Number of**  **OTU** | **Number of species** | **Number of genera** | **Number of families** | **Number of**  **order** | **Number of**  **class** | **Number of phylum** | **Coverage** |
| --- | --- | --- | --- | --- | --- | --- | --- | --- | --- |
| BK | 61476 | 2162 | 885 | 459 | 256 | 154 | 62 | 26 | 0.989 |
| RSH | 60870 | 2231 | 981 | 509 | 272 | 161 | 63 | 26 | 0.988 |
| RSL | 67832 | 2343 | 991 | 508 | 275 | 162 | 65 | 26 | 0.989 |
| RSN | 65125 | 2043 | 822 | 421 | 245 | 149 | 64 | 27 | 0.989 |
| RZH | 56103 | 2367 | 1051 | 543 | 296 | 172 | 68 | 30 | 0.988 |
| RZL | 63197 | 2240 | 947 | 488 | 265 | 156 | 61 | 26 | 0.987 |
| RZN | 64168 | 1965 | 830 | 429 | 249 | 153 | 64 | 26 | 0.989 |

**Table S3** General features of the fungal community sequencing results in soil samples

| **Sample**  **ID** | **Number of sequences** | **Number of**  **OTU** | **Number of species** | **Number of genera** | **Number of families** | **Number of**  **order** | **Number of**  **class** | **Number of phylum** | **Coverage** |
| --- | --- | --- | --- | --- | --- | --- | --- | --- | --- |
| BK | 64537 | 689 | 299 | 213 | 136 | 66 | 28 | 10 | 0.998 |
| RSH | 58983 | 708 | 314 | 219 | 138 | 65 | 29 | 11 | 0.997 |
| RSL | 64684 | 667 | 307 | 218 | 135 | 65 | 28 | 11 | 0.997 |
| RSN | 62761 | 665 | 294. | 208 | 130 | 64 | 26 | 9 | 0.997 |
| RZH | 67577 | 662 | 297 | 213 | 134 | 66 | 30 | 11 | 0.997 |
| RZL | 67781 | 653 | 287 | 207 | 133 | 64 | 28 | 11 | 0.998 |
| RZN | 59853 | 631 | 276 | 198 | 131 | 65 | 28 | 11 | 0.998 |

**Table S4** Alpha indexes in the bacterial community of all samples

| **Samples** | **Ace** | **Chao** | **Shannon** | **Simpson** | **Sobs** |
| --- | --- | --- | --- | --- | --- |
| BK | 2847.3±87.28ab | 2836.2±63.13ab | 5.98±0.065a | 0.0063±0.00048a | 2161.7±90.60ab |
| RSN | 2631.2±97.09b | 2636.3±92.50ab | 5.92±0.049a | 0.0067±0.00041a | 2043.0±38.19ab |
| RSL | 2996.1±177.65ab | 2975.5±159.19ab | 6.07±0.052a | 0.0061±0.00030a | 2343.0±165.00ab |
| RSH | 2881.6±233.88ab | 2862.3±277.78ab | 6.02±0.214a | 0.0066±0.00142a | 2231.0±222.69ab |
| RZN | 2600.8±81.09b | 2626.6±49.47b | 5.89±0.020a | 0.0067±0.00016a | 1964.7±36.12b |
| RZL | 2964.1±105.92ab | 2952.2±96.90ab | 5.95±0.014a | 0.0071±0.00028a | 2239.7±77.22ab |
| RZH | 3061.4±167.63a | 3046.1±178.06a | 6.05±0.185a | 0.0064±0.00109a | 2367.0±218.75a |

**Table S5** Alpha indexes in the fungal community of all samples

| **Samples** | **Ace** | **Chao** | **Shannon** | **Simpson** | **Sobs** |
| --- | --- | --- | --- | --- | --- |
| BK | 819.1±1.60a | 831.5±11.14a | 3.72±0.238a | 0.0731±0.02354a | 689.3±10.30a |
| RSN | 815.2±32.69a | 816.7±40.88a | 3.54±0.168a | 0.1081±0.00396a | 665.3±22.55a |
| RSL | 835.7±43.10a | 841.3±56.30a | 3.42±0.325a | 0.1270±0.04627a | 667.3±32.59a |
| RSH | 838.3±71.79a | 840.6±78.38a | 3.98±0.150a | 0.0601±0.01400a | 708.3±57.71a |
| RZN | 764.1±90.27a | 757.3±83.05a | 3.24±0.531a | 0.1462±0.06483a | 631.0±79.78a |
| RZL | 800.5±68.31a | 793.9±61.97a | 3.47±0.260a | 0.1026±0.03108a | 653.3±44.39a |
| RZH | 796.1±26.00a | 799.4±44.06a | 3.40±0.264a | 0.1315±0.04711a | 662.3±8.38a |

**Table S6** Effects of extract of the tuberous root of *P. heterophylla* inoculated with *F. oxysporum* on the growth of the differential isolates

| Isolate id | Species | Fold Change | p_value | enrich |
| --- | --- | --- | --- | --- |
| H1-3-G4 | *Aeromonas veronii* | 0.374054 | 0.036302 | promote |
| L2-3-C7 | *Aeromonas veronii* | 0.348412 | 0.041284 | inhibit |
| L2-3-D1 | *Aeromonas veronii* | 0.332471 | 0.035553 | inhibit |
| L2-3-D7 | *Aeromonas hydrophila* | 0.792245 | 0.010588 | inhibit |
| L2-3-E4 | *Aeromonas veronii* | 0.484939 | 0.000575 | inhibit |
| L2-3-G7 | *Aeromonas veronii* | 1.277974 | 0.031582 | inhibit |
| L3-3-A4 | *Aeromonas hydrophila* | 0.457617 | 0.035766 | inhibit |
| L3-3-B1 | *Aeromonas hydrophila* | 3.348612 | 0.011622 | inhibit |
| L3-3-C1 | *Aeromonas hydrophila* | 4.356565 | 0.001889 | promote |
| N2-3-F7 | *Aeromonas enteropelogenes* | 2.010271 | 0.030359 | promote |
| N1-3-B4 | *Enterobacter asburiae* | 0.320328 | 0.043589 | promote |
| N3-3-F1 | *Flavobacterium* sp. | 3.187741 | 2.59E-03 | promote |
| H2-3-D7 | *Lactococcus lactis* | 0.415701 | 0.036723 | promote |
| N1-3-A1 | *Myroides marinus* | 0.357721 | 0.036081 | inhibit |
| H4-3-D1 | Others | 0.416108 | 0.030112 | promote |
| H1-3-A7 | *Pseudomonas poae* | 0.340441 | 0.013373 | promote |
| H2-3-A7 | *Pseudomonas poae* | 0.877494 | 9.78E-06 | promote |
| H4-3-C1 | *Pseudomonas poae* | 0.367087 | 0.027097 | promote |
| N3-3-C4 | *Pseudomonas poae* | 0.74077 | 0.039143 | promote |
| N1-3-C7 | *Serratia odorifera* | 0.495631 | 0.000751 | promote |
| H1-3-B1 | *Sphingobacterium anhuiense* | 2.239797 | 6.07E-06 | inhibit |
| H1-3-C4 | *Sphingobacterium* sp. | 0.367152 | 0.031313 | promote |
| H2-3-E1 | *Stenotrophomonas* sp. | 0.40118 | 0.044957 | promote |
| L2-3-F1 | *Stenotrophomonas* sp. | 3.986794 | 8.03E-05 | inhibit |
| N1-3-D4 | *Stenotrophomonas rhizophila* | 2.727825 | 0.03213 | promote |
| N1-3-G4 | *Stenotrophomonas rhizophila* | 1.726588 | 0.002018 | inhibit |

**Table S7** Specific qPCR primers designed for absolute quantification of *F. oxysporum* and *P. poae*

| **Target species** | **Primer** | **Sequence (5' - 3')** | **Gene** | **Reference** |
| --- | --- | --- | --- | --- |
| *Fusarium* | ITS1-F | CTTGGTCATTTAGAGGAAGTAA | *ITS* | (Lievens et al., 2005) |
|  | AFP308R | CGAATTAACGCGAGTCCCAA |  |  |
| *Pseudomonas* | sucDF | CCAGGCGAATGCAAGATCGGCATCA | *sucD* | (Zhang et al., 2019) |
|  | sucDR | GCTTCTTCTTCAGCCGAACCGCCGA |  |  |

**Table S8** Numbers of significantly (*P* < 0.05) differentiated bacterial genera between groups evaluated by left/right-tailed Wilcoxon test (observed 811 genera)

|  | **RSH** | **RSL** | **RSN** | **RZH** | **RZL** | **RZN** | **BK** |
| --- | --- | --- | --- | --- | --- | --- | --- |
| **RSH** |  | 24/22  5.67% | 80/37  14.43% | 15/16  3.82% | 20/17  4.56% | 64/21  10.48% | 61/41  12.58% |
| **RSL** |  |  | 84/21  12.95% | 33/39  8.75% | 37/25  7.64% | 101/22  15.17% | 90/36  15.54% |
| **RSN** |  |  |  | 98/44  17.51% | 71/32  12.70% | 18/16  4.19% | 36/14  6.17% |
| **RZH** |  |  |  |  | 30/18  5.92% | 88/22  13.56% | 80/35  14.18% |
| **RZL** |  |  |  |  |  | 62/17  9.74% | 62/28  11.10% |
| **RZN** |  |  |  |  |  |  | 32/9  5.06% |
| **BK** |  |  |  |  |  |  |  |

**Table S9** Numbers of significantly (*P* < 0.05) differentiated fungal genera between groups evaluated by left/right-tailed Wilcoxon test (observed 446 genera)

|  | **RSH** | **RSL** | **RSN** | **RZH** | **RZL** | **RZN** | **BK** |
| --- | --- | --- | --- | --- | --- | --- | --- |
| **RSH** |  | 14/4  4.04% | 34/13  10.54% | 17/6  5.16% | 27/6  7.40% | 27/3  6.44% | 30/17  10.09% |
| **RSL** |  |  | 12/22  7.62% | 10/13  5.16% | 13/8  4.71% | 22/16  8.15% | 16/20  7.73% |
| **RSN** |  |  |  | 17/26  9.64% | 13/22  7.51% | 19/6  5.36% | 14/8  4.72% |
| **RZH** |  |  |  |  | 9/5  3.00% | 13/8  4.51% | 15/14  6.22% |
| **RZL** |  |  |  |  |  | 8/7  3.22% | 8/19  5.79% |
| **RZN** |  |  |  |  |  |  | 17/5  4.93% |
| **BK** |  |  |  |  |  |  |  |

**Reference**

Lievens B, Brouwer M, Vanachter AC, Lévesque CA, Cammue BP, Thomma BP. Quantitative assessment of phytopathogenic fungi in various substrates using a DNA macroarray. Environ Microbiol 2005;7(11):1698-710.

Zhang J, Li N, Wu Q, Gu Q, Zhang Y, Cai S, Bai J. A characteristic nucleotide sequence and a specific primer, kit and detection method for detecting Pseudomonas2019.
